# Restrictive versus conventional ward fluid therapy in non-cardiac surgery patients and the effect on postoperative complications: a meta-analysis

**DOI:** 10.1186/s13741-023-00337-9

**Published:** 2023-09-21

**Authors:** Joachim J. Bosboom, Marije Wijnberge, Bart F. Geerts, Martijn Kerstens, Michael G. Mythen, Alexander P. J. Vlaar, Markus W. Hollmann, Denise P. Veelo

**Affiliations:** 1 Department of Anesthesiology, Amsterdam UMC, Amsterdam University, Amsterdam, the Netherlands; 2 Department of Intensive Care Medicine, Amsterdam UMC, Amsterdam University, Amsterdam, the Netherlands; 3 Department of Anesthesiology, Intensive Care, and Pain Medicine, Amphia Hospital, Breda, The Netherlands; 4 Healthplus.Ai-R&D B.V., Amsterdam, The Netherlands; 5 Departments of Anesthesia and Critical Care, University College London Hospitals, National Institute of Health Research Biomedical Research Centre, London, UK

**Keywords:** Fluid therapy, Postoperative period, Infusions, Intravenous

## Abstract

**Background:**

Diligent fluid management is an instrumental part of Enhanced Recovery After Surgery. However, the effect of a ward regimen to limit intravenous fluid administration on outcome remains unclear. We performed a meta-analysis investigating the effect of a restrictive versus a conventional fluid regimen on complications in patients after non-cardiac surgery in the postoperative period on the clinical ward.

**Study design:**

We performed a systematic search in MEDLINE, Embase, Cochrane Library, and CINAHL databases, from the start of indexing until June 2022, with constraints for English language and adult human study participants. Data were combined using classic methods of meta-analyses and were expressed as weighted pooled risk ratio (RR) or odds ratio (OR) with 95% confidence interval (CI). Quality assessment and risk of bias analyses was performed according to PRISMA guidelines.

**Results:**

Seven records, three randomized controlled trials, and four non-randomized studies were included with a total of 883 patients. A restrictive fluid regimen was associated with a reduction in overall complication rate in the RCTs (RR 0.46, 95% CI 0.23 to 0.95; *P* < .03; *I*^2^ = 35%). This reduction in overall complication rate was not consistent in the non-randomized studies (RR 0.74, 95% CI 0.53 to 1.03; *P* 0.07; *I*^2^ = 45%). No significant association was found for mortality using a restrictive fluid regimen (RCTs OR 0.51, 95% CI 0.05 to 4.90; *P* = 0.56; *I*^2^ = 0%, non-randomized studies OR 0.30, 95% CI 0.06 to 1.46; *P* = 0.14; *I*^2^ = 0%). A restrictive fluid regimen is significantly associated with a reduction in postoperative length of stay in the non-randomized studies (MD − 1.81 days, 95% CI − 3.27 to − 0.35; *P* = 0.01; *I*^2^ = 0%) but not in the RCTs (MD 0.60 days, 95% CI − 0.75 to 1.95; *P* = 0.38). Risk of bias was moderate to high. Methodological quality was very low to moderate.

**Conclusion:**

This meta-analysis suggests restrictive fluid therapy on the ward may be associated with an effect on postoperative complication rate. However, the quality of evidence was moderate to low, the sample size was small, and the data came from both RCTs and non-randomized studies.

**Supplementary Information:**

The online version contains supplementary material available at 10.1186/s13741-023-00337-9.

## Introduction

Currently, one in every four patients undergoing surgery suffer from one or more complications (Eappen et al. 2013). Besides the burden of complications on patients, the economic impact is overwhelming. Complications increase length of hospital stay and treatment cost up to 150% (Khan et al. 2006).

Hypervolemia as well as hypovolemia has been shown to lead to major complications, such as pneumonia and anastomotic leakage after surgery, and can even contribute to death (Chappell et al. 2008). Fluid balance is known as an independent predictor of outcome after surgery in several studies, (Thacker et al. 2016; Shin 2018) especially in colorectal surgery (Brandstrup et al. 2003; MacKay et al. 2006). Over time fluid regimens have shifted from liberal to restrictive, including goal-directed fluid therapy (GDT), to limit positive perioperative fluid balances (Chappell et al. 2008; Pearse et al. 2014; Holte and Kehlet 2006). In GDT, stroke volume (SV) or cardiac output (CO) is optimized by titrating fluid and cardiovascular stimulants. The goal is to keep the patient normovolemic and to prevent hyper- or hypovolemia. Although more extensive studies are still needed, current evidence suggests that intraoperative GDT decreases morbidity after major surgery (Som et al. 2017; Rollins and Lobo 2016). Diligent fluid management is an instrumental part of Enhanced Recovery After Surgery (ERAS) pathways (Gustafsson et al. 2013). However, it is hard to assess the impact of each item of the ERAS bundle separately (Jurt et al. 2017).

Implementation of GDT is a time-intensive and financially costly investment. Although patients spent most of the time on the ward, application of fluid optimization strategies are mostly limited to theatres, post-anesthesia care units (PACU), and intensive care units (ICU). Therefore, a reduction of the amount of fluid given to patients on the ward might contribute substantially to outcome. This systematic review aims to meta-analyze the available evidence for a restrictive fluid regimen on the ward.

## Methods

We performed a systematic review and meta-analysis following the Preferred Reporting Items for Systematic Reviews and Meta-analyses (PRISMA) guidelines (Moher et al. 2009). Details of the protocol for this systematic review were registered on PROSPERO and are accessible athttp://www.crd.york.ac.uk/PROSPERO/display_record.php?ID=CRD42017075304.

### Search strategy

With support from a clinical librarian, a search in MEDLINE, Embase, Cochrane Library, and CINAHL databases was performed from the start of indexing until June 2022, with constraints for English language and adult human study participants. Duplicate studies were excluded. The full-search strategy is available on PROSPERO (see details regarding the search strategy in Additional file 1).

### Study selection

The following eligibility criteria were specified: (1) randomized controlled trials (RCTs) and non-randomized studies, (2) the studied population should include adult patients undergoing elective non-cardiac surgery in the postoperative period on the ward (≥ 2 h after surgery and ≤ 30 days) without further restrictions for type of anesthesia, (3) the intervention should include a restrictive fluid regimen (solely or as part of an ERAS protocol) compared to a conventional fluid regimen, and (4) the studies should report on the incidence of complications. Studies investigating fluid regimens at high care units (e.g., ICU or PACU) were excluded.

Title and abstract of the records were screened for relevance with the use of a systematic review system (Rayyan, Data Analytics (QCRI), Doha, Qatar) (Ouzzani et al. 2016). This system allows for a blinded screening by two independent reviewers (JB and MK). After identification of the records, reference lists were screened for additional relevant records. Two reviewers independently reviewed the full-text records and selected relevant studies based on the inclusion criteria (JB and MK). Discrepancies were resolved by discussion with a third reviewer (MW).

Primary outcome was overall complications. Complications, morbidity, or adverse events were defined as overall complications. Secondary outcomes extracted were the postoperative length of hospital stay (PLOS), mortality (30-day, 90-day, hospital or overall mortality), and severe complications (≥ grade 3 using the Clavien-Dindo classification (Dindo et al. 2004) or major complications/major adverse events).

### Data synthesis and analysis

Data were extracted from the articles and appendices by two independent reviewers (JB and MW) using a standardized form. This form was an adapted version of the Data Extraction and Assessment Template from the Cochrane Public Health Group. The variables extracted included the year of publication, type of study, country, number of patients, type of patients, type of surgery, type of hospital, the age of patients, gender, ASA score, the aim of the study, the definition of complications used, the complications looked at in the study, the definition of restricted and conventional fluid regimen, and outcomes reported as mentioned above.

### Risk of bias

RCTs were assessed for the risk of bias according to a ten-point checklist (Cochrane). Risk of bias due to missing results in the synthesis was assessed according the Cochrane Handbook Sect. 13.3.3 (Higgins 2008). For non-randomized studies, the Newcastle–Ottawa Scale (NOS) was used to evaluate the risk of bias (Wells 2013). A maximum of nine stars can be awarded. The quality of the body of evidence was assessed employing the Grades of Recommendation, Assessment, Development and Evaluation (GRADE) approach (Guyatt et al. 2011).

For each study, the risk ratio (RR) for common events (i.e., > 20%) or Peto odds ratio (OR) method for rare events, and the 95% confidence intervals for complications and mortality comparing the restrictive and the standard groups were calculated (Deeks et al. 2005). For PLOS, the mean (SD) or median (IQR) was extracted, and SD was estimated with Wan’s method when not given (Wan et al. n.d.). In the protocol submitted to PROSPERO we intended to use the Mantel–Haenszel method to compute a weighted-pooled odds ratio based on the fixed effects model, however, based on an expected clinical, and methodological diversity we used the random-effects model (Mantel and Haenszel 1959; Borenstein et al. 2010). For data analysis, we used a software program (RevMan, version 5.4; The Cochrane Collaboration). We performed a post hoc trial sequential analysis (TSA) to assess whether our result of this meta-analysis are mathematically supported, with the use of the TSA software package (version 0.9.5.9 beta) (Wetterslev et al. 2008). We calculated information size using O’Brien-Flemming boundaries, setting the risk of a type 1 error at one-sided 5% and power at 80%. We used the data of the included studies to calculate relative risk reduction and incidence of events in our study (RRR 53%, incidence restrictive arm 21%, incidence conventional arm 46%).

## Results

Our systematic search resulted in 4050 relevant records after removal of duplicates. Review of the titles and abstracts excluded 3986 records. After full-text review of 64 records, seven articles were included (PRISMA diagram, see Additional file 2 and 3 for exclusion criteria).

### Study characteristics

Seven studies, three RCTs, and four non-randomized studies with a total of 883 patients were included (Table 1) (de 2009; Lobo et al. 2002; Morgan et al. 2016; Muller et al. 2009; Vermeulen et al. 2009; Walsh et al. 2008; Zargar-Shoshtari et al. 2008). Data of a total of 859 patients were included in this meta-analysis, because of exclusion of patients in the original studies. The studies were published between 2002 and 2016. The studies focused on major gastrointestinal surgery, ranging from colorectal to pancreatic surgery. Study characteristics are shown in Table 1. The number of complications was a primary endpoint in four out of seven studies. All studies looked at infectious and non-infectious complications including wound infection, respiratory complications, and complications related to surgery such as an anastomotic leakage, postoperative ileus, and pancreatic fistula. For details regarding the definition of complication and the complications scored per study see Tables 2 and 3.

**Table 1:** Study characteristics

**Study**	**Country**	***N***	**Type of surgery**	**Restricted** **fluid regimen**^a^	**Conventional fluid regimen**^a^	**Primary outcome**
**RCTs**						
Lobo, 2002	UK	20	Hemicolectomy	2000 ml	3000 ml	Gastric emptying
Muller, 2009	Switzerland	156	Open colonic	0 ml	2000 ml	Complications
Vermeulen, 2009	Netherlands	62	Major abdominal	1500 ml	2500 ml	Postoperative LOS
**Non-randomized studies**						
de Aguilar-Nascimento, 2009	Brazil	61	Major abdominal	< 30 ml/kg	30–50 ml/kg	Complications
Morgan, 2016	USA	378	Pancreatic	1800 ml	Non-restricted	-
Walsh, 2008	UK	106	Midline laparotomy	< 3000 ml	> 3000 ml	Complications
Zargar-Shoshtari, 2008	New Zealand	100	Colonic	667 ml	2167 ml	Complications

*Legend*: *N* number of patients, *RCTs* randomized clinical trials, *LOS* length of stay

^a^Definition in amount of fluid per day

**Table 2:** Definition of complications

Study	Definition complications	Complications scored
Lobo, 2002	Infectious and non-infectious complications during the first 30 postoperative days	Wound infection, peripheral edema, vomiting on day 4, electrolyte disturbances, confusion, respiratory infection
Muller, 2009	Total 30-day complication rate, severity of complications according to Calvien-Dindo classification	Intra-abdominal abscess, wound infection, anastomotic leaks, cardiovascular events, urinary infection/retention, postoperative bleeding, pneumonia/respiratory events, postoperative ileus
Vermeulen, 2009	Predefined postoperative complications within 30 days after discharge, according National Surgical Adverse Event Registration from the Dutch Society for Surgery	Major; cardiac events, anastomotic leakage, sepsis, kidney failure requiring dialysis Minor; abdominal wound abscess, infection or dehiscence, respiratory disorders or infection, bleeding, peripheral thrombo-embolism
de Aguilar-Nascimento, 2009	Number of complications	Surgical site infection, anastomotic dehiscence, pulmonary, sepsis, shock
Morgan, 2016	Overall complication rate	Including wound infection, pneumonia and pancreatic fistula rates
Walsh, 2008	Any predefined complication	Cardiac arrest, myocardial infarction, unstable angina, cerebrovascular accident, respiratory failure, pneumonia, pulmonary embolus, deep venous thrombosis, prolonged ileus, anastomotic leak, urinary tract infection, wound infection, abscess formation, gastrointestinal bleed
Zargar-Shoshtari, 2008	Predefined complications, well-documented that required specific interventions	Ileus, urinary tract infection, wound infection, chest infection, fluid overload, cardiac, urinary retention, anastomotic leak

**Table 3:** Summary of findings

Outcomes	Illustrative comparative risks* (95% CI)		Relative effect (95% CI)	No of participants (studies)	Quality of the evidence (GRADE)
	**Assumed risk**	**Intervention risk/risk difference**			
	**Conventional regimen**	**Restrictive regimen**			
**Complications** Overall rate	**Randomized controlled trials**		**RR 0.46** (0.23 to 0.95)	214 patients (3 studies)	⊕ ⊕ ⊕ ⊝ Moderate^1,2^
	**455 per 1000**	**210 per 1000** (105 to 432 per 1000)			
	**Non-randomized studies**			645 patients (4 studies)	⊕ ⊕ ⊝ ⊝ Low
	**585 per 1000**	**433 per 1000** (310 to 602 per 1000)	**RR 0.74** (0.53 to 1.03)		
**Mortality**	**Randomized controlled trials**		**OR 0.51** (0.05 to 4.90)	214 (3 studies)	⊕ ⊕ ⊝ ⊝ Low^2^
	**2 per 1000**	**1 fewer per 1000** (0 fewer to 10 fewer per 1000)			
	**Non-randomized studies**				⊕ ⊕ ⊝ ⊝ Low
	**16 per 1000**	**5 fewer per 1000** (4 fewer to 22 fewer per 1000)	**OR 0.30** (0.06 to 1.46)	537 patients (3 patients	
**Postoperative length of stay (PLOS)** Scale: days	**Randomized controlled trials**				
	The mean PLOS ranged across control groups from **7 to 7** days	The mean PLOS in the intervention groups was **0.60 higher** (0.75 lower to 1.95 higher)	**MD 0.60** (0.75 to 1.95)	62 patients (1 study)	⊕ ⊝ ⊝ ⊝ Very low^3^
	**Non-randomized studies**				
	The mean PLOS ranged across control groups from **7 to 12** days	The mean PLOS in the intervention groups was **1.81 lower** (3.27 lower to 0.35 lower)	**MD − 1.81** (**− **3.27 to − 0.35)	439 patients (2 studies)	⊕ ⊝ ⊝ ⊝ Very low^3^

*CI* Confidence interval, *OR* Odds Ratio, *MD* Mean Difference

^*^Assumed risk: mean baseline risks of the studies. The Risk difference (and its 95% confidence interval) is based on the assumed risk in the conventional fluid regimen group and the relative effect of the intervention (and its 95% CI)

^1^High risk for reporting bias in 3 out of 4 studies. One study terminated prematurely

^2^One included randomized trial terminated prematurely

^3^Inconsistency not explained. Therefore, we downgraded by one scale starting from low

### Fluid regimens

Intraoperative fluid regimens were similar (de 2009; Lobo et al. 2002; Morgan et al. 2016; Vermeulen et al. 2009) or differed only minimal between the conventional group and restricted group in the studies (Muller et al. 2009). Intraoperative fluid balance significantly differed in one study (Zargar-Shoshtari et al. 2008) of those not describing the intraoperative fluid regimen (Additional file 4) (Walsh et al. 2008; Zargar-Shoshtari et al. 2008). No data for fluid balances were available.

Postoperative conventional fluid therapy ranged between more than two liters a day and non-restricted. The intervention groups were treated with zero to less than 3 l a day. The exact fluid regimens are stated in Table 1. The type of postoperative fluid administered was saline, Ringer’s lactate, and dextrose.

### Risk of bias

Risk of bias was high for reporting bias in the three RCTs (see Additional file 5). For non-randomized studies risk of bias was moderate (3–6 stars on NOS, see Additional file 5). Risk of bias due to missing results in a synthesis was assessed. Results are available in Additional file 5. There was no mortality assessed for the separate cohorts in the study of Walsh (Walsh et al. 2008). For the other studies, there were no missing results for synthesis.

### Outcomes

**Fig. 1:**
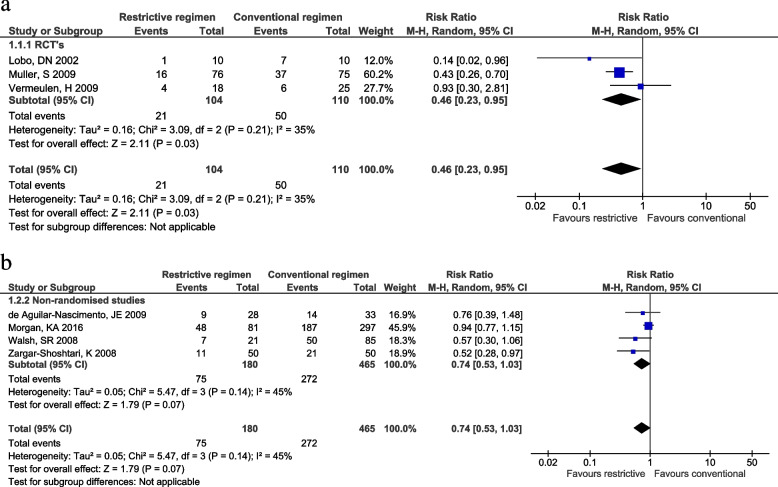
**a** Forest plot restrictive fluid regimen versus conventional fluid regimen in RCTs for outcome complications. **b** Forest plot restrictive fluid regimen versus conventional fluid regimen in NRS for outcome complications

**Fig. 2:**
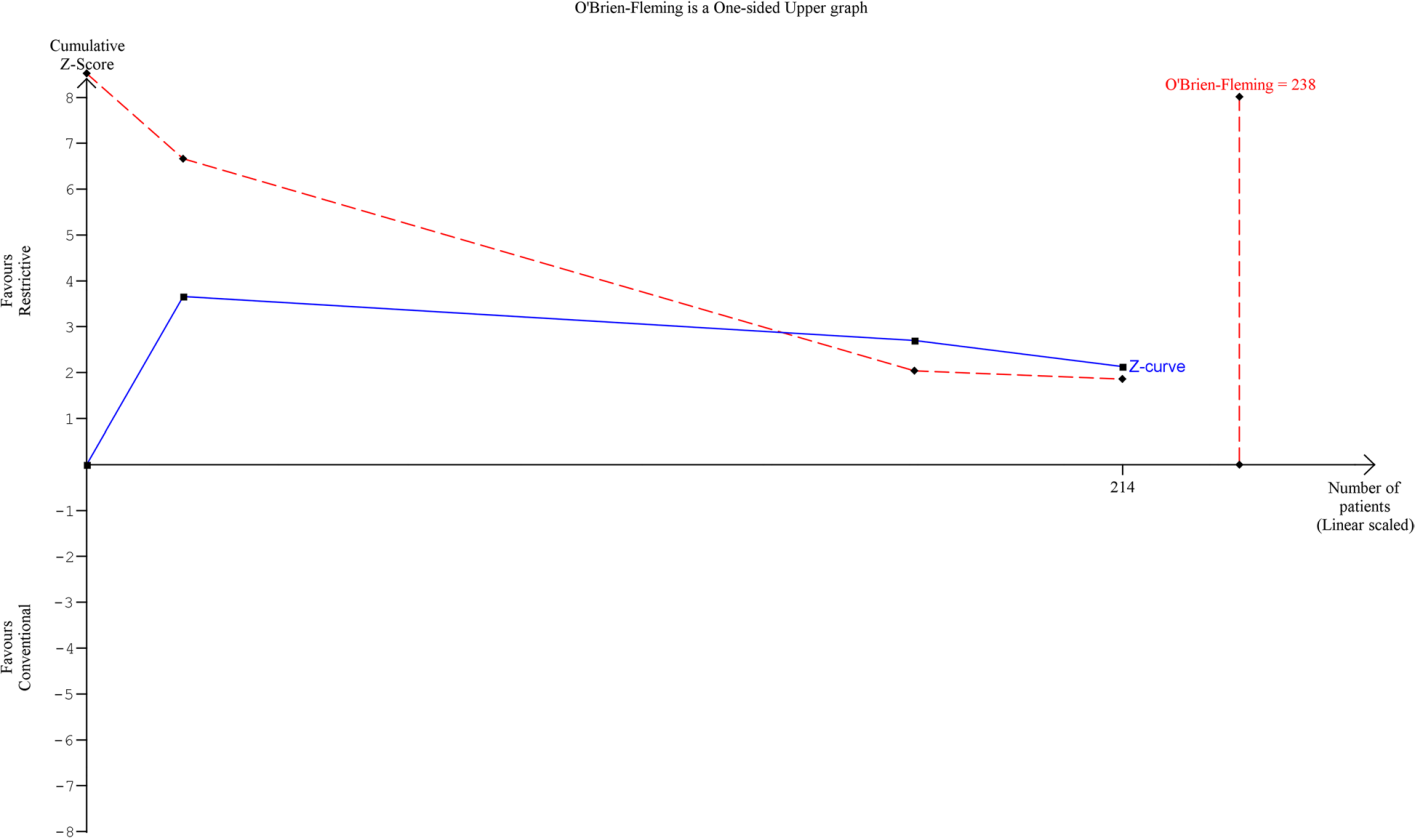
Trial sequential analysis. Legend: The TSA showing the cumulative *Z* score (blue line) crossing the 5% trial sequential monitoring boundaries (red dashed inward-sloping line). The heterogeneity-adjusted information size is 238 (vertical red dashed line)

A restrictive fluid regimen is associated with a reduction in overall complication rate in RCTs (RR 0.46, 95% CI 0.23 to 0.95; *P* < 0.03; *I*^2^ = 35%) (Fig. 1a). This reduction in overall complication rate was not statistically significant in the non-randomized studies (RR 0.74, 95% CI 0.0.53 to 1.03; *P* 0.07; *I*^2^ = 45%) (Fig. 1b). Mortality was defined as 30-day (Lobo et al. 2002), 90-day (Morgan et al. 2016), in-hospital (Vermeulen et al. 2009; Walsh et al. 2008), or overall mortality (de 2009; Zargar-Shoshtari et al. 2008). A restrictive fluid regimen is not significantly associated with a reduction in mortality in the RCTs (OR 0.51, 95% CI 0.05 to 4.90;*P* = 0.56; *I*^2^ = 0%) or in the non-randomized studies (OR 0.30, 95% CI 0.06 to 1.46; *P* = 0.14; *I*^2^ = 0%) (Additional file 6). A restrictive fluid regimen is significantly associated with a reduction in PLOS in the non-randomized studies (mean difference − 1.81, 95% CI − 3.27 to − 0.35; *P* = 0.01; *I*^2^ = 0%) but not in the RCTs (mean difference 0.60, 95% CI − 0.75 to 1.95; *P* = 0.38) (see Additional file 7). Severe complications were described in two studies and defined as major complications (Vermeulen et al. 2009) or as a Clavien-Dindo ≥ grade 3 (Muller et al. 2009). Severe complications in a restrictive fluid regimen presented in 3/76 vs 7/75 patients and 1/18 vs 3/25 patients in a conventional fluid regimen. Insufficient data were available to analyze severe complications. The TSA showed the cumulative*Z* score crossed the 5% trial sequential monitoring boundaries and therefore is supportive of the meta-analysis (Fig. 2). Furthermore, TSA showed the heterogeneity-adjusted required information size to demonstrate a 53% % relative risk reduction of overall complications (with a proportion of 46% of complications in the conventional fluid regimen group, an alpha of 5%, and a beta of 20%) is 238 patients.

## Discussion

In this meta-analysis, we reviewed and compared fluid management strategies in the ward. The main finding is that a restrictive fluid regimen is significantly associated with a reduction in complications in RCTs and a reduction in PLOS in non-randomized studies. However, a restrictive fluid regimen is not significantly associated with a reduction in complications in non-randomized studies or a decrease in mortality in the under 1000 patients studied.

Restricted or patient-tailored fluid therapy is part of ERAS (Gustafsson et al. 2013). It is difficult to assess the effectiveness of each part of the ERAS bundle separately (Jurt et al. 2017). Our finding of an association between a postoperative fluid regimen to prevent hypervolemia and a reduction in complications is in line with large retrospective cohort studies, systematic reviews, and a meta-analysis (Thacker et al. 2016; Holte and Kehlet 2006; Rollins and Lobo 2016; Varadhan and Lobo 2010; Thacker et al. 2014). However, not all trials investigating restrictive fluid therapy show this association (Vermeulen et al. 2009; Srinivasa et al. 2013; van Samkar et al. 2015). One of the included RCTs was terminated prematurely because of frequent protocol violations (e.g., unblinding by personnel not involved in the trial) and insufficient patient inclusion (Vermeulen et al. 2009). Significantly more complications in the restrictive fluid regimen group in an intention-to-treat analysis were seen. Reasons for unblinding were not directly related to hypovolemia. Furthermore, after unmasking the amount of fluid given was not recorded and treatment effect was not measured. For this meta-analysis, we were interested in the effect of adhering to the intervention as described in the study. We chose to include the per-protocol analyses, because the estimation of its effects relates most closely to the implications of our analysis (Higgins 2022). This may be biased as this analysis is restricted to the patients who adhered to the study protocol.

We performed a TSA to determine if the results of this met-analysis are mathematically supported (Wetterslev et al. 2008). Based on these TSA, a required information size of 238 patients in a meta-analysis is necessary to confirm the effect of a restrictive fluid regimen on overall complications on the ward and to exclude early overestimation (Wetterslev et al. 2008). Recently, a large retrospective cohort study showed perioperative fluid volume to be an independent predictor for length of hospital stay (Aga et al. 2016). However, there was no distinction between intra- and postoperative fluid volume.

Despite several studies and the “The British Consensus Guidelines concerning postoperative fluid therapy,” there is no widely accepted appropriate fluid therapy on the ward (Powell-Tuck 2011). A wide range of what is called “restrictive” or “conventional” is presented in the included studies, with an overlap between the definitions and thereby influencing data and interpretation. A previous trial attempted to define fluid restriction as < 1.75 L per day, and liberal as > 2.75 L per day, which seems a fair summary of the averages used in literature (Varadhan and Lobo 2010). All studies included major gastrointestinal surgery, ranging from colonic resections to pancreatic operations, with different underlying conditions. Thus, the wide range of the types of surgical procedures makes the studies more heterogenic. Furthermore, only a small number of studies with a limited number of participants and heterogeneity in the primary endpoint, study design, and definition of fluid regimens could be included in the present meta-analysis. Perioperative complications were a primary endpoint in one RCT and three retrospective studies. Therefore, the included RCTs might be underpowered to assess complications. We used a random-effects model as we expect the effect size varies between studies (Borenstein et al. 2010). However, meta-analysis shows a statistical significant reduction of the complication rate in RCTs and a trend towards significance in non-randomized studies (Fig. 1). The limited number of participants may explain why the effect of a reduction in complication rate did not translate into a reduced mortality and only a decreased PLOS in non-randomized studies. Also, the complications might not be severe enough to lead to a clinically relevant reduction. We included severe complications as a secondary outcome. Only limited data were available. Therefore, we did not perform analyses on these data. Intraoperative fluid regimens were (near-)similar in the included studies, and intraoperative fluid volume was only significantly different in one retrospective study (Zargar-Shoshtari et al. 2008). Therefore, we think intraoperative fluid regimens did not influence our results in this meta-analysis. Last, it is not clear how signs of hypovolemia (e.g., hypotension or thirst) where treated in the included retrospective studies. Assessing the risk of bias resulted in a high or unclear risk of bias for some of the assessed bias domains. To address this issue, we evaluated the quality of evidence using a validated tool supported by the PRISMA statement (Moher et al. 2009; Guyatt et al. 2011). The significance level was the same for all outcomes. Therefore, there is a risk of overestimating the effect due to multiple testing. Furthermore, there is a risk of confounding by induction for the non-randomized studies. Patients may have received more fluids because they developed more complications or may have needed less fluids because they had fewer complications.

Overall, it seems that a restrictive fluid regimen may help in improving post-surgical patient outcome. Still, some challenges for prescription of fluids apply. A fluid balance is difficult to obtain (Walsh et al. 2008; Boersema et al. 2014). Studies show that weighing is often neglected and fluid charts are not registered correctly (Walsh et al. 2008; Boersema et al. 2014). Prescriptions were not based on the patient’s current status, and junior doctors ordered the majority of prescriptions as they mostly care for patients on the ward (Walsh et al. 2008; Nadler et al. 2014; Lobo et al. 2001). A small audit in 2015 showed that 60% of the patients were not treated according to protocol (Birk 2015). Following the studies mentioned earlier, only 40% of patients were weighed, and fluid charts were inaccurate. Moreover, several large-scale surveys found that protocols or guidelines for fluid therapy or even standard hemodynamic monitoring are not present in around 75% of hospitals (Holte and Kehlet 2006; Geerts 2009). Different studies suggest fewer complications arise when comparing a restricted fluid regimen to conventional therapy (MacKay et al. 2006; Lobo et al. 2002; Muller et al. 2010; Gonzalez-Fajardo et al. 2009). Our analysis shows a significantly lower risk ratio for complications with restricted use of fluids postoperatively in RCTs. We can therefore only assume that there is a serious gap in our hemodynamic assessment capabilities on the ward. Wearable technology, non-invasive cardiac output monitoring, and even sophisticated algorithms are now becoming available and could be a potential solution for this shortcoming (Michard 2017). However, currently, there are no data available to support the use of these techniques, and implementation can be expensive.

Our meta-analysis suggests that reduction of complications with more diligent fluid therapy on the ward may be feasible. Individualized fluid prescription in a more structured approach may be beneficial, based on protocols addressing both the volume status and the clinical response of the patient (Myles et al. 2017). The studies describing the effect of a restrictive fluid regimen on complication rate showed an overall significant difference in the meta-analysis. We performed a TSA for the RCT’s with primary outcome overall complications and found an information size of 238 patients to be necessary for a meta-analysis to draw more solid conclusions.

## Conclusions

Our study suggests a restrictive approach towards intravenous fluid use on the ward following major gastrointestinal surgery may be associated with a statistically significant effect on complication rate in RCTs and postoperative length of stay in non-randomized studies. However, the quality of evidence was moderate to low, the sample size was small, and the data came from both RCTs and non-randomized studies. The association did not translate into reduced mortality.

### Supplementary Information

**Additional file 1.** Search documents.

**Additional file 2.** PRISMA flow diagram.

**Additional file 3.** Full text screening—exclusion criteria.

**Additional file 4.** Intraoperative fluid regimens.

**Additional file 5.** Risk of bias summary.

**Additional file 6.** Mortality.

**Additional file 7.** PLOS.

## Data Availability

The datasets used and analyzed during the current study are available from the corresponding author on reasonable request.
