# Supplementary material for: Restrictive versus conventional ward fluid therapy in non-cardiac surgery patients and the effect on postoperative complications: a meta-analysis
Source: Perioper Med (Lond). 2023 Sep 21;12:52. doi: 10.1186/s13741-023-00337-9 (PMC10514989; doi:10.1186/s13741-023-00337-9)
Supplement: Supplementary file 1 — Additional file 1. Search documents. [file 13741_2023_337_MOESM1_ESM.doc]

# Restrictive versus conventional ward fluid therapy in non-cardiac surgery patients and the effect on postoperative complications; A Meta-analysis

With support from a clinical librarian, a search in MEDLINE, Embase, Cochrane Library, and CINAHL databases was performed from the start of indexing until June 2022, with constraints for English language and adult human study participants.

**PubMed**

(("Postoperative Period"[Mesh] OR "Postoperative Care"[Mesh] OR postoperat* [tiab] OR post-operat* [tiab]) AND ("Fluid Therapy"[Mesh] OR "Infusions, Intravenous"[Mesh] OR fluid therap*[tiab] OR rehydration[tiab] OR fluid regimen*[tiab] OR fluid management[tiab] OR postoperative intravenous fluid*[tiab]) AND ("Postoperative Complications"[Mesh] OR "Fluid Therapy/adverse effects"[Mesh] OR postoperative outcome[tiab]  OR postoperative hospital stay[tiab] OR ((postoperati*[tiab] OR treatment[tiab] OR fluid-related[tiab]) AND (outcome[tiab] OR complication*[tiab]))) AND ("Fluid Therapy/methods"[MAJR] OR management[tiab] OR program*[tiab] OR intake[tiab] OR practice[tiab] OR fluid regimen*[tiab] OR fluid restriction[tiab]) AND ("Randomized Controlled Trial"[pt] OR "Cohort Studies"[Mesh] OR retrospective[tiab] OR guideline[tiab] OR protocol[tiab] OR random*[tiab]))

**EMBASE (via Ovid):**

| **#** | **Searches** | **Results** |
| --- | --- | --- |
| 1 | postoperative period/ or postoperative care/ or (postoperat* or post-operat*).ti,ab. |  |
| 2 | exp fluid therapy/ or fluid intake/ or (fluid therap* or rehydration or fluid regimen* or fluid management or postoperative intravenous fluid*).ti,ab. |  |
| 3 | exp postoperative complication/ or fluid therapy/ae or outcome assessment/ or co.fs. or (postoperative outcome or postoperative hospital stay or ((postoperati*or treatment or fluid-related) and (outcome or complication*))).ti,ab. |  |
| 4 | procedures/ or (management or program* or intake or practice or fluid regimen* or fluid restriction).ti,ab. |  |
| 5 | randomized controlled trial/ or cohort analysis/ or major clinical study/ or priority journal/ or (retrospective or guideline or protocol or random*).ti,ab. |  |
| 6 | 1 and 2 and 3 and 4 and 5 |  |

**Nnnnn**

| **#** | **Searches** | **Results** |
| --- | --- | --- |
| 1 | postoperative period/ or postoperative care/ or (postoperat* or post-operat*).ti,ab. |  |
| 2 | exp fluid therapy/ or fluid intake/ or (fluid therap* or rehydration or fluid regimen* or fluid management or postoperative intravenous fluid*).ti,ab. |  |
| 3 | exp postoperative complication/ or fluid therapy/ae or outcome assessment/ or co.fs. or (postoperative outcome or postoperative hospital stay or ((postoperati*or treatment or fluid-related) and (outcome or complication*))).ti,ab. |  |
| 4 | procedures/ or (management or program* or intake or practice or fluid regimen* or fluid restriction).ti,ab. |  |
| 5 | randomized controlled trial/ or cohort analysis/ or major clinical study/ or priority journal/ or (retrospective or guideline or protocol or random*).ti,ab. |  |
| 6 | 1 and 2 and 3 and 4 and 5 |  |
| 7 | 6 and 2017:2017. (sa_year) |  |

**CINAHL (via Ebsco):**

( ((MH "Postoperative Care+") OR (MH "Postoperative Period") OR (TI post-operat* OR AB post-operat*) OR (TI postoperat* OR AB postoperat*)) ) AND ( ((MH "Fluid Therapy+") OR (MH "Fluid Resuscitation") OR (MH "Intravenous Therapy+") OR (MH "Oral Rehydration Therapy") OR (MH "Fluid Intake") OR (TI fluid therap* OR AB fluid therap*) OR (TI rehydration OR AB rehydration) OR (TI fluid regimen* OR AB fluid regimen*) OR (TI fluid management OR AB fluid management) OR (TI postoperative intravenous fluid* OR AB postoperative intravenous fluid*)) ) AND ( ((MH "Postoperative Complications+") OR (TI postoperative hospital stay OR AB postoperative hospital stay) OR (((TI postoperati* OR AB postoperati*) OR (TI treatment OR AB treatment) OR (TI fluid-related OR AB fluid-related)) AND ((TI outcome OR AB outcome) OR (TI complication OR AB complication))) ) AND ( ((MH "Clinical Trials+") OR (MH "Prospective Studies+") OR (TI retrospective OR AB retrospective) OR (TI guideline OR AB guideline) OR (TI protocol OR AB protocol) OR (TI random* OR AB random*) )

((MH "Postoperative Care+") OR (MH "Postoperative Period") OR (TI post-operat* OR AB post-operat*) OR (TI postoperat* OR AB postoperat*))

AND

((MH "Fluid Therapy+") OR (MH "Fluid Resuscitation") OR (MH "Intravenous Therapy+") OR (MH "Oral Rehydration Therapy") OR (MH "Fluid Intake")  OR (TI fluid therap* OR AB fluid therap*) OR (TI rehydration OR AB rehydration) OR (TI fluid regimen* OR AB fluid regimen*) OR (TI fluid management OR AB fluid management) OR (TI postoperative intravenous fluid* OR AB postoperative intravenous fluid*))

AND

((MH "Postoperative Complications+") OR (TI postoperative hospital stay OR AB postoperative hospital stay) OR (((TI postoperati* OR AB postoperati*) OR (TI treatment OR AB treatment) OR (TI fluid-related OR AB fluid-related)) AND ((TI outcome OR AB outcome) OR (TI complication OR AB complication)))

AND

((MH "Clinical Trials+") OR (MH "Prospective Studies+") OR (TI retrospective OR AB retrospective) OR (TI guideline OR AB guideline) OR (TI protocol OR AB protocol) OR (TI random* OR AB random*)

**Cochrane Library**

ID Search Hits

#1 MeSH descriptor: [Postoperative Period] this term only

#2 postoperat* or post-operat*:ti,ab,kw (Word variations have been searched)

#4 #1 or #2

#5 MeSH descriptor: [Fluid Therapy] this term only

#6 fluid therap* or rehydration or fluid regimen* or fluid management or postoperative intravenous fluid*:ti,ab,kw (Word variations have been searched)

#7 #5 or #6

#8 MeSH descriptor: [Postoperative Complications] this term only

#9 MeSH descriptor: [Fluid Therapy] explode all trees and with qualifier(s): [Adverse effects - AE]

#10 postoperative outcome or postoperative hospital stay:ti,ab,kw (Word variations have been searched)

#11 (postoperati*or treatment or fluid-related) and (outcome or complication*):ti,ab,kw (Word variations have been searched)

#12 #8 or #9 or #10 or #11

#13 MeSH descriptor: [Fluid Therapy] explode all trees and with qualifier(s): [Methods - MT]

#14 management or program* or intake or practice or fluid regimen* or fluid restriction:ti,ab,kw (Word variations have been searched)

#15 #13 or #14

#16 #4 and #7 and #12 and #15 in Cochrane Reviews (Reviews and Protocols), Other Reviews and Trials
