# Supplementary material for: Restrictive versus conventional ward fluid therapy in non-cardiac surgery patients and the effect on postoperative complications: a meta-analysis
Source: Perioper Med (Lond). 2023 Sep 21;12:52. doi: 10.1186/s13741-023-00337-9 (PMC10514989; doi:10.1186/s13741-023-00337-9)
Supplement: Supplementary file 2 — Additional file 2. PRISMA flow diagram. [file 13741_2023_337_MOESM2_ESM.doc]

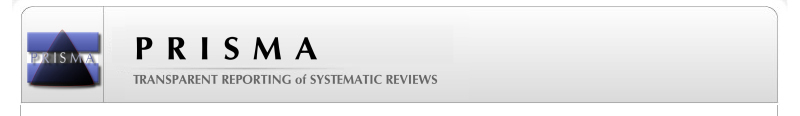
**PRISMA 2009 Flow Diagram**

**Screening**

**Included**

**Eligibility**

**Identification**

Records identified through database searching (n = 4088)

Records after duplicates removed
(n = 4050)

Records screened
(n = 4050)

Records excluded
(n = 3986)

Full-text articles assessed for eligibility
(n = 64 )

Full-text articles excluded (n = 57)

- No comparison restrictive vs conventional fluid regimen (n = 18)

- No postoperative fluid management data (n = 15)

- No data available to compute OR (n = 5)

- Wrong study design (n = 4)

- High care unit (n = 15)

Studies included in qualitative synthesis
(n = 7 )

Studies included in quantitative synthesis (meta-analysis)
(n = 7 )
