# Supplementary material for: Restrictive versus conventional ward fluid therapy in non-cardiac surgery patients and the effect on postoperative complications: a meta-analysis
Source: Perioper Med (Lond). 2023 Sep 21;12:52. doi: 10.1186/s13741-023-00337-9 (PMC10514989; doi:10.1186/s13741-023-00337-9)
Supplement: Supplementary file 4 — Additional file 4. Intraoperative fluid regimens. [file 13741_2023_337_MOESM4_ESM.doc]

| **Study** | **Country** | **N** | **Type of surgery** | **Intraoperative fluid regimen** | **Restricted**  **fluid regimen** | **Conventional fluid regimen** |
| --- | --- | --- | --- | --- | --- | --- |
| **RCT’s** |  |  |  |  |  |  |
| Lobo, 2002 | UK | 20 | Hemicolectomy | Prescribed by anesthesiologist unaware of randomization | 2000 ml | 3000 ml |
| Muller, 2009 | Switzerland | 156 | Open colonic | *Restricted* 5 mL/kg/h  *Conventional* 10 mL/kg/h | 0 ml | 2000 ml |
| Vermeulen, 2009 | Netherlands | 62 | Major abdominal | Standardized protocol for both groups | 1500 ml | 2500 ml |
|  |  |  |  |  |  |  |
| **Non-randomized trials** |  |  |  |  |  |  |
| de Aguilar-Nascimento, 2009 | Brazil | 61 | Major abdominal | Standardized protocol 10-20 mL/kg/h | < 30 ml/kg | 30 – 50 ml/kg |
| Morgan, 2016 | USA | 378 | Pancreatic | Standardized protocol (goal-directed fluid management) | 1800 ml | Non-restricted |
| Walsh, 2008 | UK | 106 | Midline laparotomy | *Restricted* standardized protocol (goal-directed fluid management)  *Conventional* not described | < 3000 ml | > 3000 ml |
| Zargar-Shoshtari, 2008 | New Zealand | 100 | Colonic | *Restricted* standardized protocol (goal-directed fluid management)  *Conventional* not described# | 667 ml | 2167 ml |

**Additional File 4** Intraoperative fluid regimens

#significantly less intraoperative fluid in the restricted fluid regimen g roup.
