# Supplementary material for: Restrictive versus conventional ward fluid therapy in non-cardiac surgery patients and the effect on postoperative complications: a meta-analysis
Source: Perioper Med (Lond). 2023 Sep 21;12:52. doi: 10.1186/s13741-023-00337-9 (PMC10514989; doi:10.1186/s13741-023-00337-9)
Supplement: Supplementary file 5 — Additional file 5. Risk of bias summary. [file 13741_2023_337_MOESM5_ESM.doc]

Additional file 5

Risk of bias summary

Risk of bias summary for randomised controlled trials (Cochrane checklist)


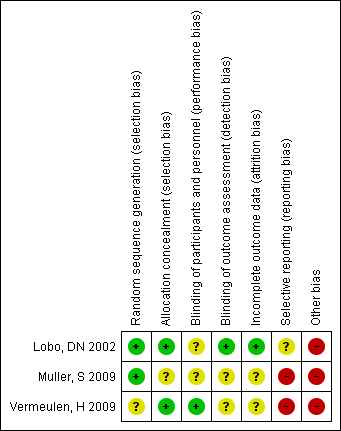


Risk of bias summary for non-randomised controlled trials (Newcastle-Ottawa Scale)

|  | **Selection** | | | | **Comparability** | | | | **Outcome** | | |  |
| --- | --- | --- | --- | --- | --- | --- | --- | --- | --- | --- | --- | --- |
| **Study** | S1 | S2 | S3 | S4 | | C1 | C2 | O1 | | O2 | O3 | Total |
| **de Aguilar-Nascimento, 2009** | * | * | * | * | |  |  |  | | * | * | 6 |
| **Morgan, 2016** | * | * | * | * | |  |  |  | | * | * | 6 |
| **Walsh, 2008** |  | * | * |  | |  |  |  | | * |  | 3 |
| **Zargar-Shoshtari, 2008** | * | * | * | * | |  |  | * | | * |  | 6 |

Table indicating availability of study results for syntheses of trials

| **Study** | **Country** | **Sample size** | **Complications** | **Mortality** | **PLOS** |
| --- | --- | --- | --- | --- | --- |
| **RCT’s** |  |  |  |  |  |
| Lobo, 2002 | UK | 20 | ✓ | ✓ | ✓ |
| Muller, 2009 | Switzerland | 156 | ✓ | ✓ | ✓ |
| Vermeulen, 2009 | Netherlands | 62 | ✓ | ✓ | ✓ |
|  |  |  |  |  |  |
| **Non-randomized studies** |  |  |  |  |  |
| de Aguilar-Nascimento, 2009 | Brazil | 61 | ✓ | ✓ | ✓ |
| Morgan, 2016 | USA | 378 | ✓ | ✓ | ✓ |
| Walsh, 2008 | UK | 106 | ✓ | ? | ? |
| Zargar-Shoshtari, 2008 | New Zealand | 100 | ✓ | ✓ | ✓ |

✓ A study result is available for inclusion in the synthesis

X No study result is available for inclusion, (probably) because the P value, magnitude or direction of the results generated were considered unfavourable by the study investigators

– No study result is available for inclusion, (probably) because the outcome was not assessed, or for a reason unrelated to the P value, magnitude or direction of the results (mortality was not reported)

? No study result is available for inclusion, and it is unclear if the outcome was assessed in the study (mortality was assessed however not for each cohort separately)
