# Supplementary material for: Restrictive versus conventional ward fluid therapy in non-cardiac surgery patients and the effect on postoperative complications: a meta-analysis
Source: Perioper Med (Lond). 2023 Sep 21;12:52. doi: 10.1186/s13741-023-00337-9 (PMC10514989; doi:10.1186/s13741-023-00337-9)

**Additional file 7**

Forrest plot for outcome postoperative length of stay in RCT’s


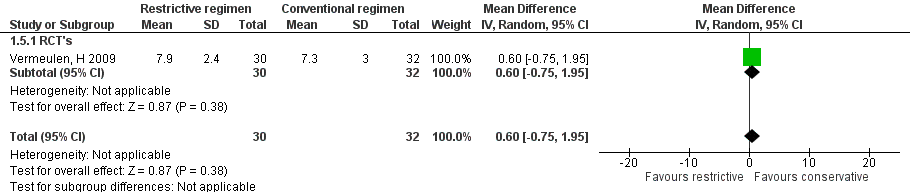


Forrest plot for outcome postoperative length of stay in non-randomized studies


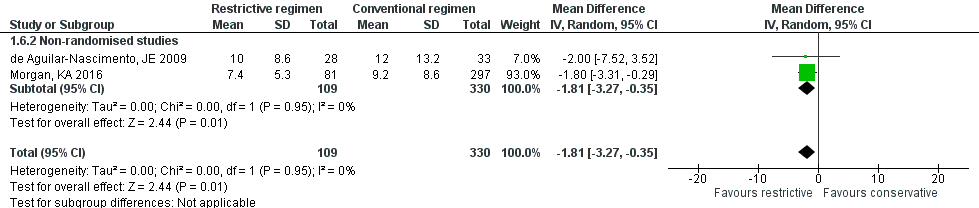

Supplement: Supplementary file 7 — Additional file 7. PLOS. [file 13741_2023_337_MOESM7_ESM.doc]
